# *α*vβ3 Integrin Antagonists Enhance Chemotherapy Response in an Orthotopic Pancreatic Cancer Model

**DOI:** 10.3389/fphar.2020.00095

**Published:** 2020-02-27

**Authors:** Melis Debreli Coskun, Thangirala Sudha, Dhruba J. Bharali, Serap Celikler, Paul J. Davis, Shaker A. Mousa

**Affiliations:** ^1^ Pharmaceutical Research Institute, Albany College of Pharmacy and Health Sciences, Rensselaer, NY, United States; ^2^ Department of Biology, Faculty of Arts and Sciences, Uludag University, Bursa, Turkey; ^3^ Department of Medicine, Albany Medical College, Albany, NY, United States

**Keywords:** pancreatic cancer, NF-κB, cisplatin, αvβ3 integrin receptor antagonist, peripheral neuropathy, motor dysfunction

## Abstract

Pancreatic cancer decreases survival time and quality of life because of drug resistance and peripheral neuropathy during conventional treatment. This study was undertaken to investigate whether αvβ3 integrin receptor antagonist compounds NDAT and XT199 can suppress the development of cisplatin resistance and cisplatin-induced peripheral neuropathy in an orthotopic pancreatic SUIT2-luc cancer cell mouse model. Anticancer effects of these compounds and their combination with cisplatin were assessed in this tumor mouse model with bioluminescent signaling and histopathology, and a cytokine assay was used to examine expression of inflammatory cytokines IL-1β, IL-6, IL-10, and TNF-α from plasma samples. To determine the neuroprotective effects of the compounds on cisplatin-induced peripheral neuropathy, behavioral hind-limb posture of the mice was evaluated. The combination therapy of NDAT or XT199 with cisplatin elicited greater inhibition of tumor growth and increased tumor necrosis compared to cisplatin alone. NDAT and XT199 in combination with cisplatin significantly decreased expression of pro-inflammatory cytokines IL-1β, IL-6, and TNF-α and significantly increased expression of anti-inflammatory cytokine IL-10 in comparison to cisplatin alone. Cisplatin-treated groups showed stocking-glove hind-limb posture, whereas NDAT and XT199 with cisplatin-treated groups displayed normal hind-limb posture. Results clearly suggest that NDAT and XT199 treatment with cisplatin that inactivates NF-κB may contribute to increased antitumor and anti-inflammatory efficacy as well as alleviate cisplatin-mediated loss of motor function in this pancreatic tumor mouse model.

## Introduction

Pancreatic cancer is a lethal malignancy, with a 5-year survival rate of only 9%. This is due to lack of diagnosis at an early stage of tumor development, ineffective therapy, its highly invasive and metastatic nature, and development of chemoresistance (Siegel et al., 2019). Drug resistance is a major reason for the inadequate efficacy of most pancreatic cancer therapies (Long et al., 2011; Mezencev et al., 2016). Cis-diamminedichloroplatinum (II), or cisplatin, a commonly used platinum-based anticancer drug for a wide range of solid tumors, is effective alone (Mezencev et al., 2016) or in combination with other chemotherapy drugs for the treatment of advanced or metastatic pancreatic cancer (Gresham et al., 2014; Ramfidis et al., 2014). Although cisplatin displays a broad spectrum of anticancer activity *via* multiple mechanisms, its clinical effectiveness is often limited due to chemoresistance and adverse side effects, especially peripheral sensory neurotoxicity (Florea and Büsselberg, 2011; Argyriou et al., 2014; Avan et al., 2015).

Cisplatin-induced peripheral neuropathy involves the hind- and upper-limbs and includes mixed signs of sensory and motor dysfunction, loss of vibration sense, loss of position sense, paresthesia, weakness, loss of taste, and tremor (Starobova and Vetter, 2017). Multiple mechanisms involved in pathophysiology of cisplatin-induced neuropathy are linked to oxidative stress, DNA damage, mitochondrial dysfunction, activation of apoptotic pathways, dysregulation of calcium homeostasis, altered ion channels activity, axonal degeneration, and loss of peripheral sensory neurons, immune processes, and neuro-inflammation (Starobova and Vetter, 2017; Zajaczkowska et al., 2019). Cisplatin was shown to kill cancer cells and primary sensory neurons in a dorsal root ganglion by a similar mechanism of apoptosis (Gill and Windebank, 1998).

Chemoresistance in pancreatic cancer is triggered by multiple mechanisms including mutations in key genes, aberrant gene expression, and deregulation of key signaling pathways. These include nuclear factor-kappaB (NF-κB), Wnt/β-catenin, Notch, Sonic Hedgehog, STAT3, PI3K/Akt, Smad/TGF-β and apoptosis pathways, epithelial–mesenchymal transition (EMT), increased angiogenesis, the presence of cancer stem cells, stroma cells and highly resistant cells, and hypoxic microenvironment inside the tumor (Long et al., 2011; Wang et al., 2011; Karandish and Mallik, 2016).

NF-κB is an important transcription factor that controls many genes involved extensively in inflammation, cancer (Hoesel and Schmid, 2013), and chemoresistance (Godwin et al., 2013). Preclinical models have demonstrated that chemotherapy drugs including cisplatin promote the activation of the NF-κB pathway, which is responsible in part for drug resistance in carcinoma cell lines (Chuang et al., 2002; Yeh et al., 2002; Yeh et al., 2003; Li et al., 2005). Cisplatin induces oxidative stress and inflammation *via* reactive oxygen species-related NF-κB pathway, implicated in peripheral neuropathy that emerges as a dose-limiting side effect (Morgan and Liu, 2011; Marullo et al., 2013; Areti et al., 2014; Vyas et al., 2014). The NF-κB pathway contributes to cancer cell development/progression and drug resistance in pancreatic cancer by inhibiting cancer cell apoptosis and inducing expression of inflammatory cytokines (Fujioka et al., 2003; Prabhu et al., 2014; Yu and Kim, 2014). These cytokines, such as interleukin-1β (IL-1β), IL-6, IL-8, IL-10, tumor necrosis factor-α (TNF-α), and transforming growth factor-β (TGF-β) are potential prognostic biomarkers as well as targets in the pathogenesis of pancreatic cancer (Fujioka et al., 2003; Prabhu et al., 2014) and in peripheral nerve injury (Fregnan et al., 2012; Wang et al., 2012; Lees et al., 2017).

Integrins are important in various cell types that affect tumor progression, especially tumor growth, angiogenesis, metastasis (Desgrosellier and Cheresh, 2010), resistance to chemotherapy (Aoudjit and Vuori, 2012), and crosstalk with growth factor receptors (Mousa et al., 2008). They are therefore attractive targets for cancer therapy. Among integrins, αvβ3 is important during tumor angiogenesis (Liu et al., 2008), and it activates several NF-κB-regulated gene expressions that are important for angiogenesis and inflammation (Chen et al., 2015). High expression of integrin αvβ3 on tumor blood vessels and some tumor cells makes it a suitable marker for cancer-targeted delivery of potential cancer therapeutics (Liu et al., 2008). In addition, integrin αvβ3 receptor is highly expressed in the plasma membrane of cancer cells and of activated endothelial cells, where it transduces thyroid hormone signals into angiogenic response and tumor cell proliferation (Bergh et al., 2005; Davis et al., 2011). Thus, the use of integrin αvβ3 antagonists and integrin-targeted delivery systems have potential as effective anti-angiogenic and anticancer therapeutics (Kumar, 2003; Hsu et al., 2007; Liu et al., 2008; Davis et al., 2014a; Davis et al., 2014b) for pancreatic cancer therapy (Chuang et al., 2013). With regard to the peripheral nervous system, integrins play a role in its development, axonal growth, Schwann cell-axon unit formation, and myelination; therefore, altered expression or function of integrins is associated with degenerative, inflammatory, and malignant disorders of this system (Previtali et al., 2001). They are therefore potential targets for the pathogenesis of chemotherapy-induced peripheral neuropathies (Dina et al., 2004; Berti et al., 2006; Antonacopoulou et al., 2010), rheumatoid arthritis related diseases (Wilder, 2002), and several neurological disorders (Wu and Reddy, 2012).

To overcome chemoresistance and reduce peripheral neurotoxicity, integrin receptor antagonists that inhibit key metabolic pathways are a promising approach to use in combination treatment for pancreatic cancer. Consequently, targeting NF-κB that is involved in the resistance of pancreatic cancer cells to cisplatin (Dizon et al., 2005; Godwin et al., 2013; Tamburrino et al., 2013) with integrin αvβ3 receptor antagonists might have great potential for development of novel preventive or therapeutic agents to overcome resistance and alleviate peripheral neuropathy in cisplatin-mediated pancreatic cancer therapies.

In this work we examined the potent and specific αvβ3 integrin antagonists Nano-diamino-tetrac (NDAT) and XT199 in an orthotopic pancreatic tumor mouse model to evaluate their efficacy on cisplatin-induced chemoresistance and peripheral neurotoxicity presenting as deficits in motor function that develop in a glove-and-stocking distribution in the hands and feet. NDAT, ({4-[4-(3-(3-(poly-2-(2-hydroxyacetotoxy))propanamido)aminopropoxy)-3,5-diiodophenoxy]-3,5-diiodophenyl} acetic acid), is a 150–200 nm poly(lactic-co-glycolic acid) (PLGA) nanoparticle covalently bound *via* a diaminopropane linker to tetraiodothyroacetic acid (tetrac) (Sudha et al., 2017a), which is a naturally occurring deaminated analog of L-thyroxine (T4). It has been shown to block the binding of thyroxine to integrin αvβ3 and the actions of the major intracellular agonist form of thyroid hormone, 3,3′,5-triiodo-L-thyronine (T3) (Davis et al., 2011; Davis et al., 2016). The NDAT formulation prohibits tetrac from entering cells and thus concentrates its activity at integrin αvβ3. Thus NDAT was formulated for targeted anticancer drug delivery and for reduced systemic toxicity (Sudha et al., 2017a). Previous studies from our laboratory have demonstrated that NDAT is an effective anticancer and anti-angiogenic agent *in vitro* and *in vivo* in the chick chorioallantoic membrane (CAM) and human cancer cell implanted mouse tumor xenograft models (Yalcin et al., 2009; Yalcin et al., 2010a; Yalcin et al., 2010b; Mousa et al., 2012; Bharali et al., 2013; Sudha et al., 2017a; Sudha et al., 2017b; Li et al., 2019). XT199, [3-(3-(3-(4, 5-dihydroimidazol-2-ylamino) propyloxylisoxazol-5-yl) carbonylamino)-2-(phenylsulfonylamino) propionic acid], is a small molecule, non-peptide selective integrin αvβ3 receptor antagonist (Bishop et al., 2001) that is an effective anti-angiogenic agent in the CAM model (Mousa et al., 2006; Bridoux et al., 2011). This anti-angiogenic effect is associated with tumor regression of human tumor cell xenografts transplanted onto the CAM by inducing apoptosis of angiogenic blood vessels (Brooks et al., 1994).

## Results

### NDAT and XT199 Promote Antitumor Effect of Cisplatin

**Figure 1:**
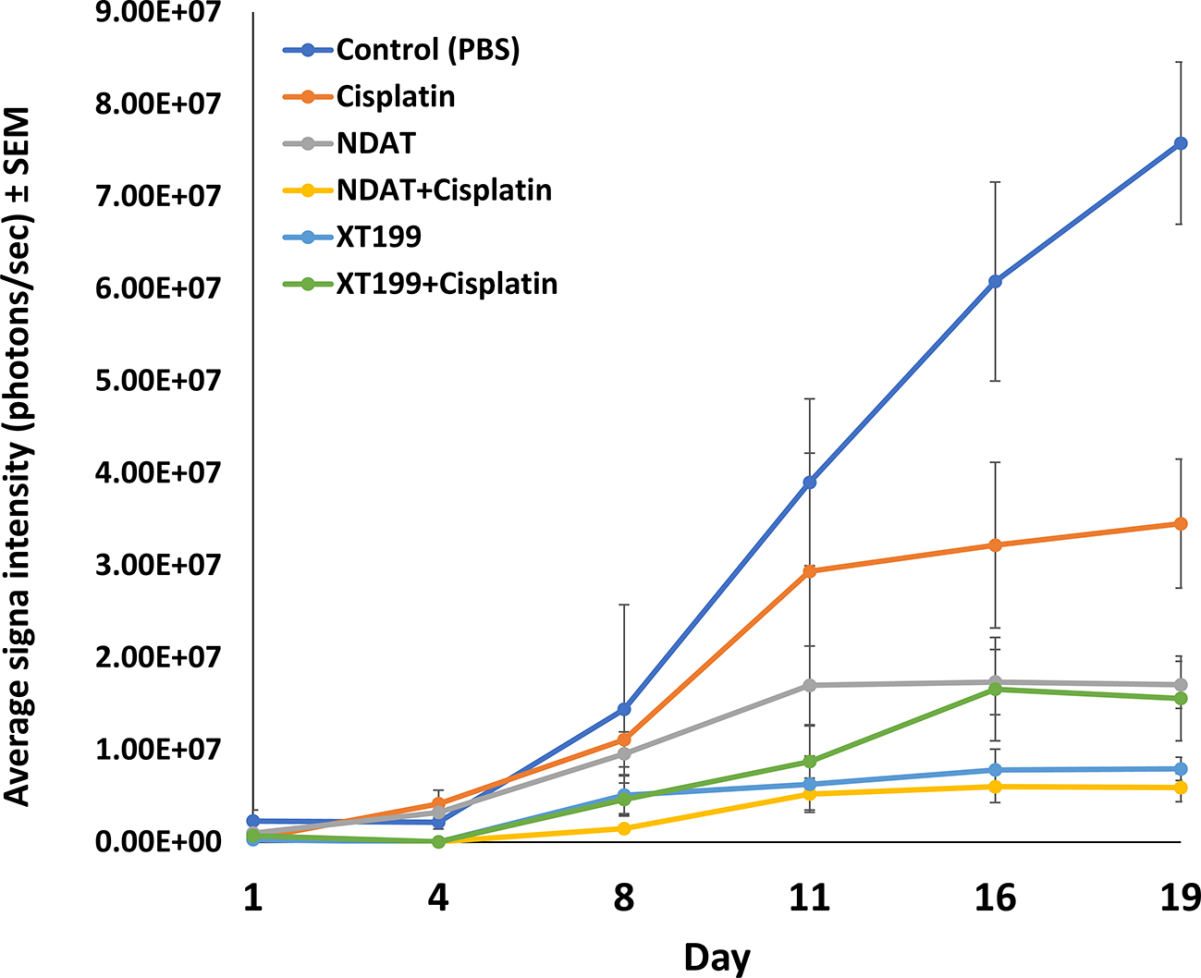
Tumor growth dynamics and signal intensity over time. Luminescent signals of SUIT2-luc orthopic tumors showing the average signals from day 1 through day 19 post-treatment. Effects of cisplatin, NDAT, XT199, and combination treatments on tumors' cancer cell viability as observed with IVIS imaging of tumor bioluminescence intensity. Data represents mean ± SEM.

**Figure 2:**
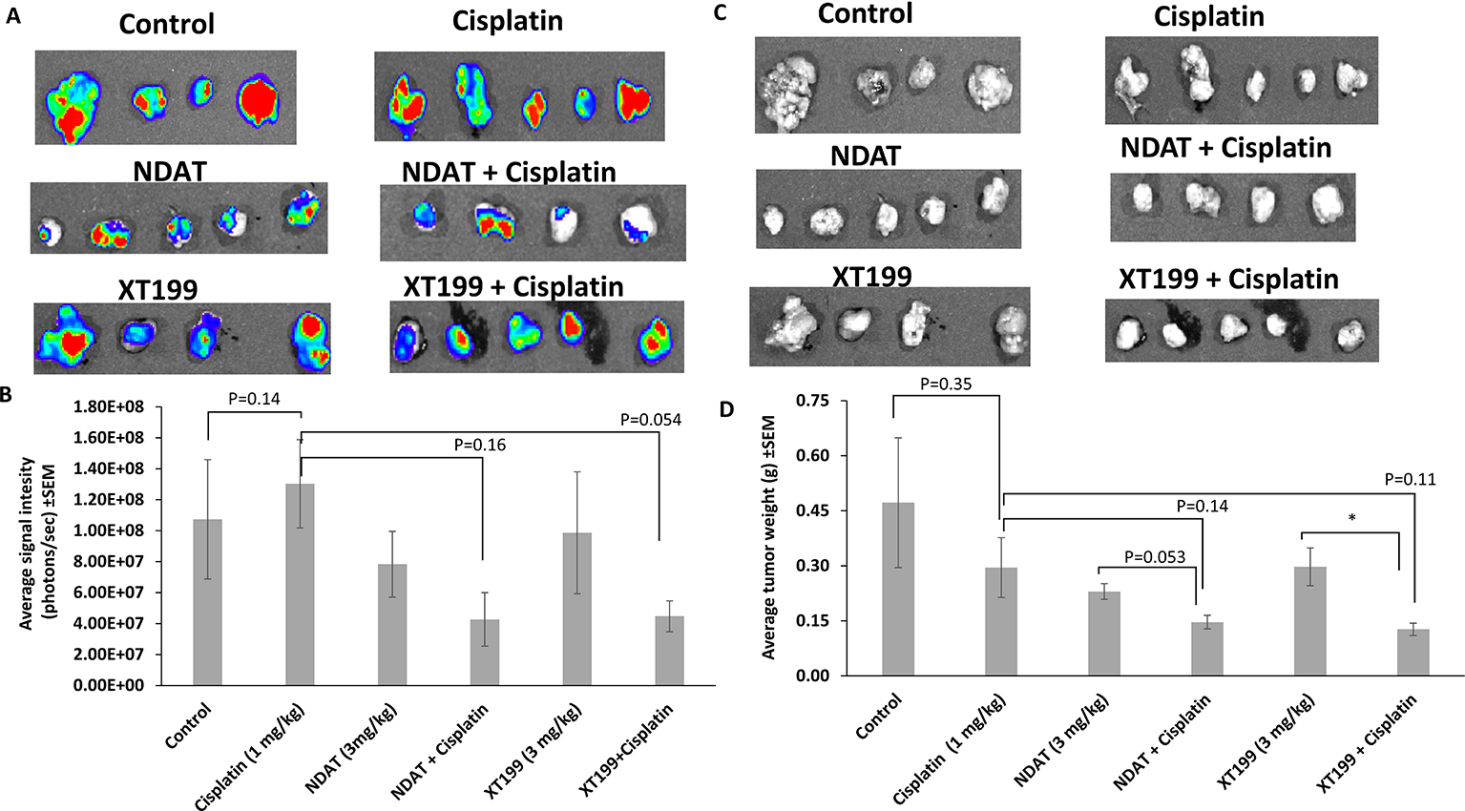
Effects of cisplatin, NDAT, XT199, and combination treatments on SUIT2-luc cancer cell viability and weight in tumors at study conclusion (day 21). **(A)** Bioluminescence images of excised orthotopic tumors of SUIT2-luc cells bearing luciferase gene. **(B)** Average signal intensity of SUIT2-luc cells for NDAT + cisplatin and XT199 + cisplatin treatments showing reduction compared to cisplatin. Image color estimates viability, ranging from nonviable (blue) to fully viable (red). **(C)** Photographic images of excised orthotopic tumors of SUIT2-luc cells bearing luciferase gene. **(D)** Tumors weight of SUIT2-luc cell orthotopic pancreatic tumors. Data represent mean ± SEM, (*p < 0.05).

NDAT and XT199 alone or in combination with cisplatin (**Table 1**) resulted in suppression of tumor signal intensity and tumor weights at 21 days following initiation of SUIT2-luc orthotopic pancreatic tumors. In contrast, control group tumors showed increased bioluminescent signal intensities with increased tumor weight over time (**Figure 1**), as observed in the *ex vivo* imaging of excised tumors (**Figures 2A, B**). It is intriguing to notice that the viable tumor cell signal intensity in cisplatin-treated animals was greater than the intensity from the control group (**Figure 2A**), yet the cisplatin-treated group had a 37.4% decrease in tumor weight compared to the control group (**Figures 2C, D**).

**Table 1:** Daily treatments with NDAT, XT199, cisplatin, and combination for the 21-day study along with number of animals per group and dose and administration route.

Treatment	Number of animals^1^	Dose (mg/kg b.w.)	Administration
Control (PBS)	4	–	s.c.
Cisplatin	5	1	i.p.
NDAT	5	3	s.c.
NDAT + Cisplatin	4	3 1	s.c. i.p.
XT199	4	3	s.c.
XT199 + Cisplatin	5	3 1	s.c. i.p.

1 Three animals were excluded from randomization into groups 2 days after initiation of SUIT2-luc orthotopic pancreatic tumors where tumor signal intensity was not detectable with IVIS, resulting in 3 groups having 4 instead of 5 animals. i.p., intraperitoneal; s.c., subcutaneous.

NDAT had a reduced signal intensity compared to control of 27%, and NDAT had an antitumor effect on tumor weight (51.3% *vs.* control). NDAT + cisplatin reduced tumor signal intensity by 60.2% and tumor weight by 69% in comparison to control. The reduction of tumor cell viability measured with IVIS revealed a clear trend in advantage of NDAT + cisplatin over NDAT and cisplatin alone treatments. NDAT + cisplatin-treated groups showed a decrease in tumor weight by 50.4% (**Figures 2C, D**) and in tumor signal intensity by 67.3% in comparison to cisplatin-treated group (**Figures 2A, B**).

XT199 had an antitumor effect on tumor weight (37% *vs.* control), and the reduction of tumor signal intensity compared to control was 8.1%. In comparison to control, XT199 + cisplatin reduced tumor weight by 73% and tumor signal intensity by 58.3%. XT199 + cisplatin-treated groups showed a decrease in tumor signal intensity by 65.6% and tumor weight by 57% when compared to the cisplatin-treated group (**Figures 2A–D**).

### NDAT and XT199 Did Not Affect Body Weight

**Figure 3:**
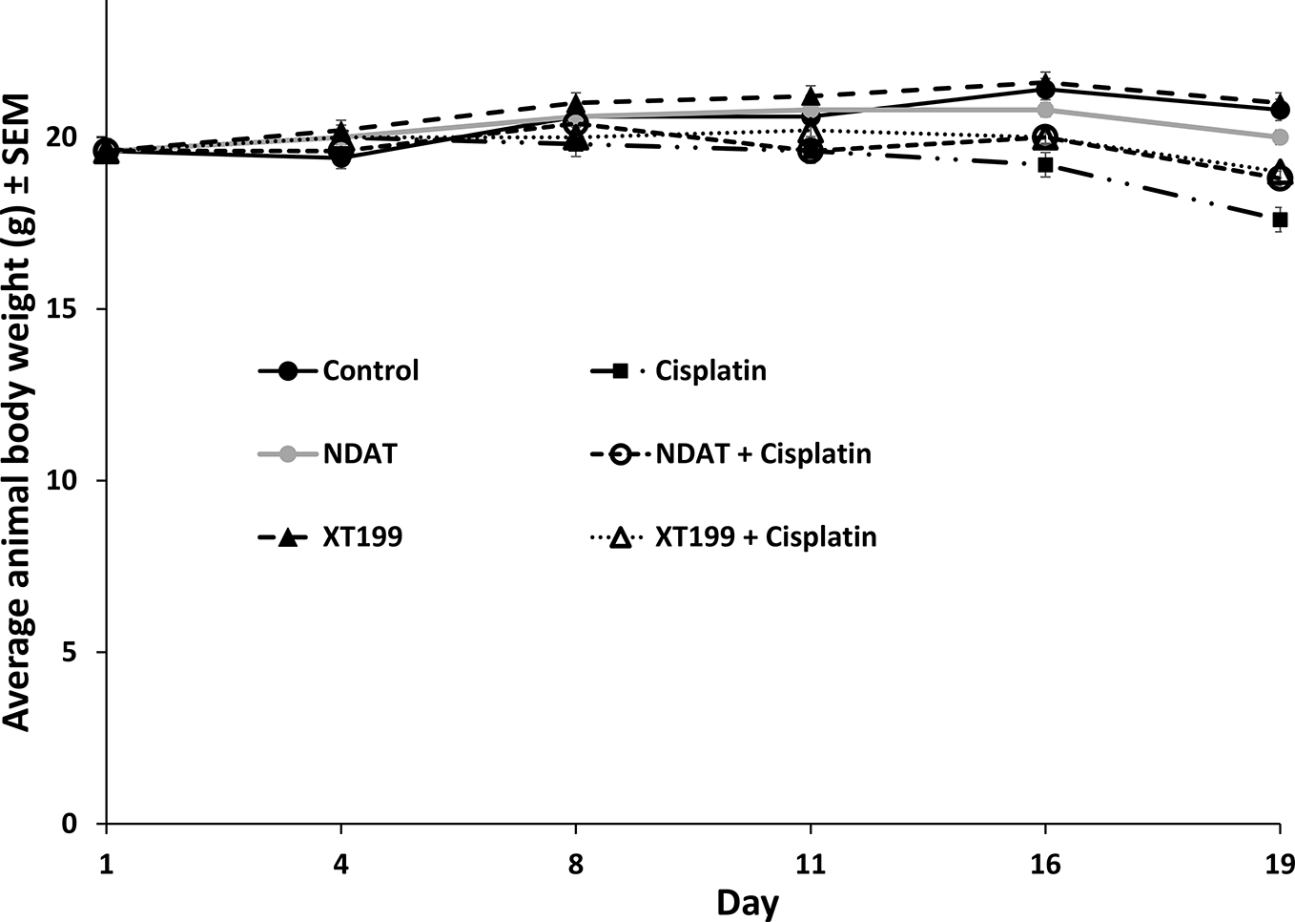
Effect of daily treatment with cisplatin vs XT199 + cisplatin or NDAT + cisplatin on body weight of mice vs control mice treated with PBS. Data represents mean ± SEM.

Body weight gain did not differ in the NDAT and XT199, alone, treatment groups in comparison to the control group from day 1 to the end of the study, but all cisplatin treatment groups had lower body weight compared to the control group. Cisplatin-treated body weights were lower after the second week and continued to decrease until the end of the experiment. This change was not statistically significant for the 21-day duration of the study (**Figure 3**). However, there is a clear trend of body weight loss on cisplatin that was reversed by either XT199 or NDAT as shown in **Figure 3**.

### Histopathological Analysis: NDAT and XT199 Alone and in CombinationWith Cisplatin Promote SUIT2-luc Tumor Necrosis

**Figure 4:**
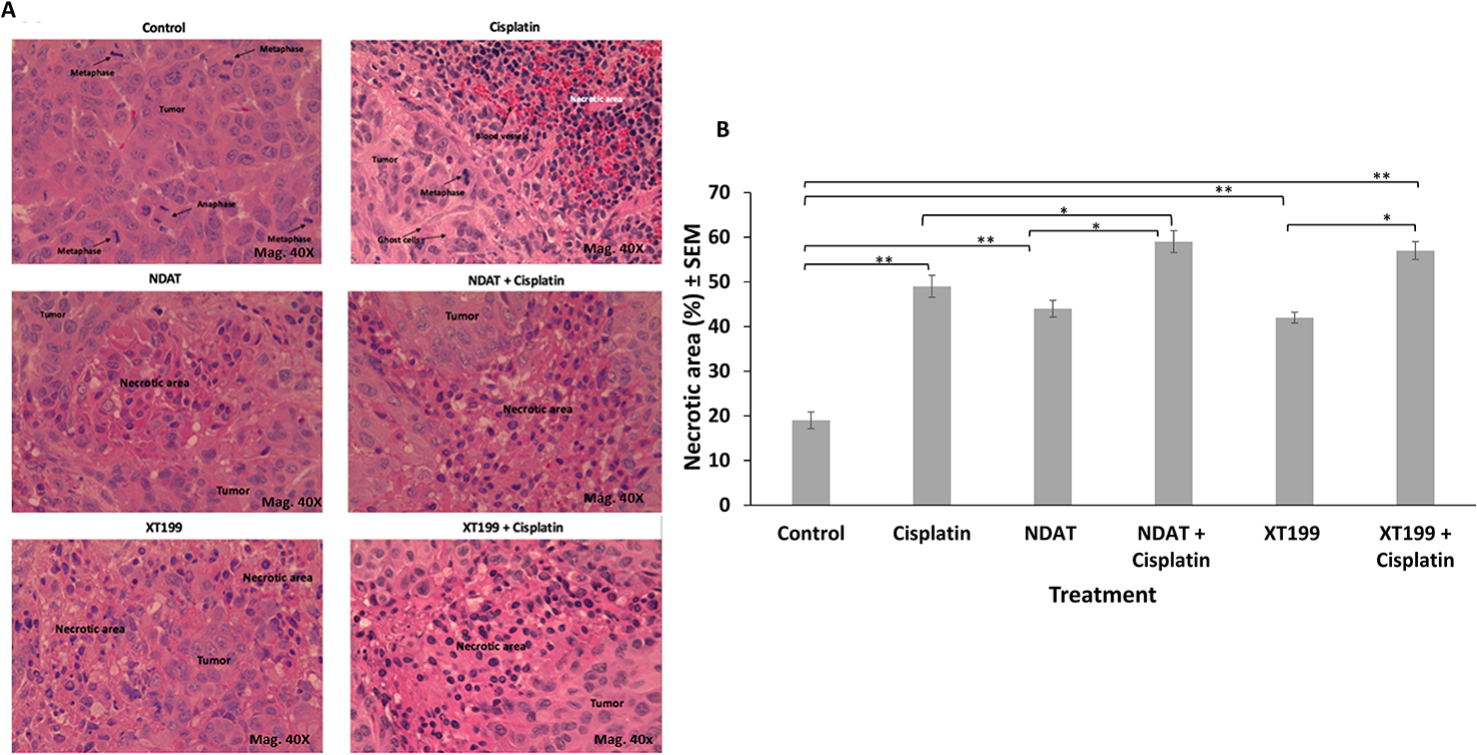
**(A)** Representative micrographs (40X) of H and E stained histological sections of orthotopic pancreatic tumors showing increased necrotic areas after treatment with NDAT and XT199 compared to control (untreated) tumor with viable cells and large nuclei at 40X. **(B)** Histopathological analysis of the orthotopic pancreatic tumors of SUIT2-luc cells treated with cisplatin showed significant increase in necrotic areas compared to control (*p < 0.05, **p < 0.01).

Cisplatin treatment resulted in significant increase in necrotic areas as compared to control (**Figures 4A, B**). Necrotic areas included fragmented and small nucleus (early stage) and ghost cells without nucleus (late stage), suggesting that cisplatin had effects on both stages of cell death. Bioluminescent signal in the tumor was inversely proportional to tumor necrosis. The treatments with NDAT and XT199 showed a significant increase in the necrotic areas (**p < 0.01) compared to control. NDAT + cisplatin (*p < 0.05) and XT199 + cisplatin-treated tumors showed large regions of necrosis when compared to cisplatin alone (**Figure 4B**).

### Inhibition of NF-κB by NDAT and XT199 Exerts Protective Effects on Cisplatin-Mediated Loss of Motor Function

**Figure 5:**
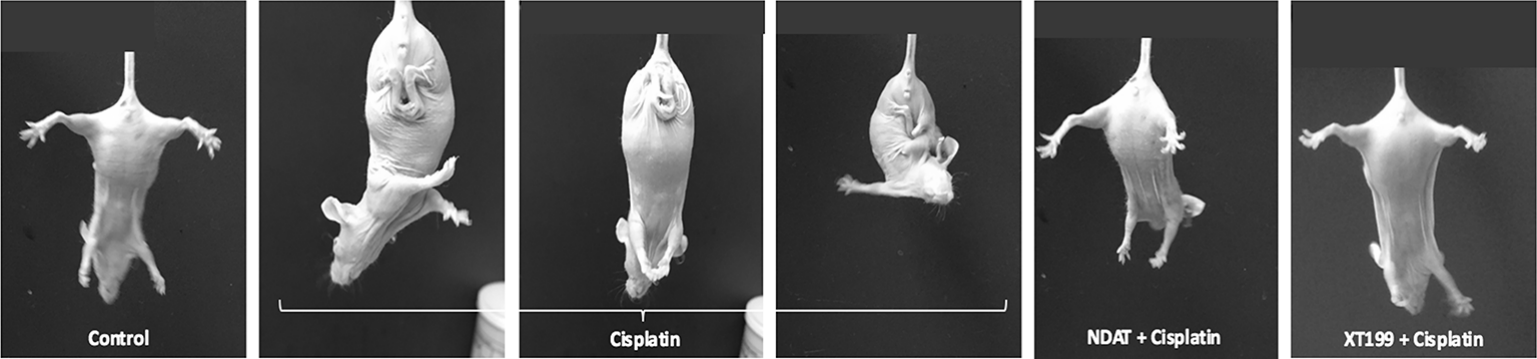
Hind-limb posture at 21 days of cisplatin treatment with and without NDAT and XT199 treatment, showing one control mouse and representative treated mice; result were the same in all treated mice. Control mouse had normal escape extension with its hind-limb upon elevation by the tail. A mouse from the cisplatin group held its hind-limbs in a crossed position close to its body as a sign of muscle weakness and motor deficits that started at day 8. Mice treated with NDAT and XT199 in combination with cisplatin did not show hind-limb posture behavior at 21-days study. The result was the same behavior with NDAT alone and XT199 alone (data not shown).

Normally, mice show normal splaying of the hind-limbs when suspended by the tail. This behavior was absent in cisplatin-treated animals, which manifested flaccidity of the hind-limbs and crossing of the limbs (**Figure 5**). Postural evaluation for motor symptoms of peripheral neuropathy revealed cisplatin-induced muscle weakness manifested by crossed hind-limbs close to the body when suspended by their tails, starting at day 8. This was an indication of stocking-glove pattern neuropathy caused by cisplatin. Control groups, NDAT + cisplatin, and XT199 + cisplatin-treated groups displayed normal motor behaviors. This provides evidence that these compounds may preserve functional motor integrity compared to cisplatin treatment alone by preserving nerve structure.

### NDAT and XT199 Downregulate IL-1β, TNF-*α*, and IL-6 Mediated Activation of NF-κB

**Figure 6:**
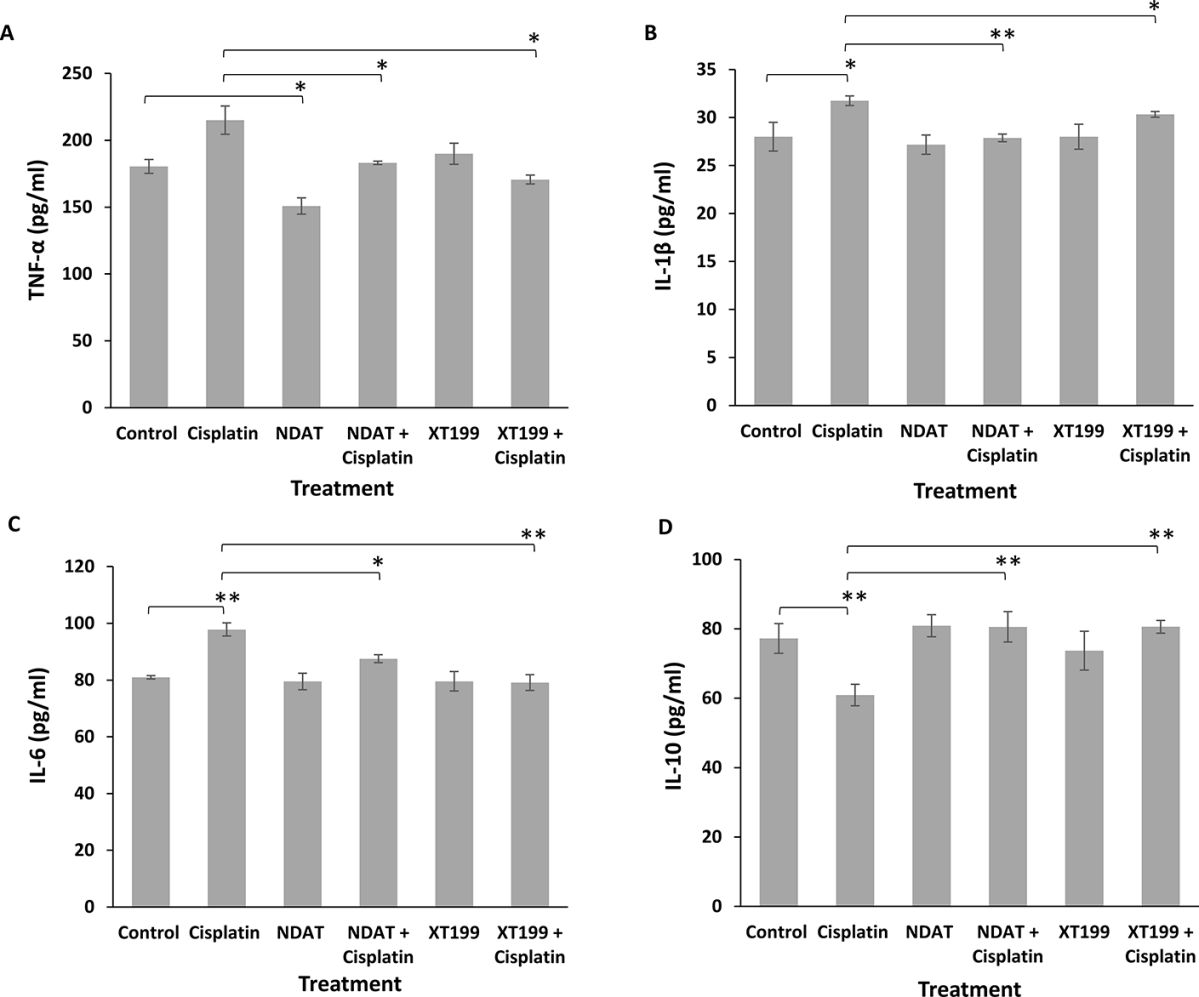
Changes in **(A)** TNF-*α* **(B)** IL-1β **(C)** IL-6 **(D)** IL-10 mean plasma concentrations for NDAT, XT199, cisplatin, and combination treated mice groups. Mice were pre-treated with NDAT (3 mg/kg) and XT199 (3 mg/kg) and post-treated with cisplatin (1 mg/kg) for 21 days. Plasma was collected to measure the concentration of these cytokines. From each treatment group, n = 4, plasma samples were tested in duplicate. Results are presented as the mean ± SEM, *p < 0.05, **p < 0.01.

Because cytokines from innate immune cells (TNF, IL-1, IL-6, IL-10, *etc*.) are usually released in small amounts, have very short half-lives, and are restricted to the tissues, we detected the changes in inflammatory cytokine responses from plasma samples of mice with SUIT2-luc orthotopic pancreatic tumors (**Figure 6**). Relative to the control group, the cisplatin-treated group exhibited increased IL-1β, TNF-α and IL-6 levels and decreased levels of IL-10. The TNF-α levels were significantly decreased by NDAT compared to control (*p < 0.05) and by the combinatorial treatments compared to cisplatin (*p < 0.05). The IL-6 and IL-1β, levels were significantly decreased by the combinatorial treatments compared to cisplatin (*p < 0.05 and **p < 0.01). The combinatorial treatments reversed the effects of cisplatin and significantly increased the IL-10 levels compared to cisplatin (**p < 0.01). In summary, any increase in these pro-inflammatory cytokines by cisplatin was effectively decreased by NDAT and XT199, and any decrease by cisplatin was effectively increased by NDAT and XT199 (**Figure 6**).

## Discussion

In cancer treatment, recognition of chemotherapy-induced peripheral neurotoxicity is important because subsequent drug discontinuation or dose adjustment may prevent further neurologic injuries. Cisplatin-induced peripheral neurotoxicity is implicated in the induction of oxidative stress and inflammatory response *via* reactive oxygen species-related NF-κB signaling pathway (Chtourou et al., 2015; Sharawy et al., 2015). Our previous study demonstrated that NDAT suppressed cisplatin-induced NF-κB activation in contrast to the NF-κB inhibitor QNZ in HeLa/NF-κB-luc reporter cells (Sudha et al., 2017a). These data suggest a greater effect of NDAT on limiting cisplatin-induced NF-κB activation.

In earlier studies we found NDAT delivery of cisplatin to urinary bladder cancer xenografts showed a 5-fold enhancement of tumor content of drug compared to conventionally administered cisplatin (Sudha et al., 2017a), and in related work we showed the efficiency of NDAT delivery of doxorubicin and paclitaxel to breast cancer and pancreatic cancer orthotopic nude mouse models, respectively; intra-tumoral concentrations of doxorubicin and paclitaxel were 2.3- and 5-fold, respectively, higher than *via* the conventional route of the administration of these agents and increased drug antitumor efficacy (Sudha et al., 2017b). The multifold increase in the tumor content of each of these widely used anticancer drugs supported the cancer-targeting ability of NDAT (Davis et al., 2011).

We show here the anti-inflammatory and anticancer actions of αvβ3 integrin antagonists NDAT and XT199 as a new approach to treat cisplatin-resistant pancreatic cancer cells. The most significant improvement in tumor response was observed with NDAT + cisplatin and XT199 + cisplatin compared to cisplatin alone in orthotopic pancreatic tumor mouse models. NDAT and XT199 alone also had an antitumor effect on pancreatic tumor signal intensity and tumor weight when compared to control (Sudha et al., 2017c; Chang et al., 2018; Li et al., 2019). Doses of NDAT and XT199 were based on previous studies with NDAT or XT199 at 1, 3, and 10 mg/kg s.c., where 10 mg/kg resulted in maximal response. In the present study, NDAT or XT199 were used at sub-maximal dose of 3 mg/kg s.c. with or without cisplatin. In contrast to our previously reported study (Sudha et al., 2017a) where NDAT acts as a targeted delivery strategy for cisplatin into tumor and its microenvironment, this study examined the effects of cisplatin combined with NDAT or XT199 and we demonstrate enhanced anti-cancer efficacy.

We previously reported on the molecular mechanisms of NDAT where it enhances pro-apoptosis genes and suppresses tumor survival genes (Chin et al., 2019).

The greater tumor bioluminescence signal intensity in cisplatin-treated animals compared to control animals indicates lack of any effect of cisplatin on the growth of the SUIT2-luc orthotopic tumors. A possible explanation could be the ability of the SUIT2-luc cells to resist cisplatin therapy. Pancreatic cancer is highly resistant to chemotherapy that is largely associated with constitutive activation of NF-κB (Long et al., 2011), and cisplatin is known to induce drug resistance in carcinoma cell lines *via* regulating NF-κB pathway (Chuang et al., 2002; Yeh et al., 2002; Yeh et al., 2003; Li et al., 2005). Taken together, NDAT and XT199 treatment enhanced the antitumor efficacy of cisplatin, resulting in the sensitization of SUIT2-luc cells to cisplatin, suggesting that these agents may target NF-κB pathway or multiple drug resistance mechanisms. These results strongly suggest that the combination of cisplatin with either NDAT or XT199 has a synergistic antitumor effect in SUIT2-luc cells.

On the other hand, cisplatin resistance in different pancreatic cancer cells was found to display significant differences in gene expression profiles (Mezencev et al., 2016). Miller et al. reported that drug resistance in pancreatic cancer was associated with the presence of multidrug resistance-associated protein (MRP) (Miller et al., 1996). Noma et al. examined the expression of MRPs (MRP1, MRP2, and MRP3) and analyzed the correlation between MRP2 expression and cisplatin resistance in human pancreatic cancer. They found that the expression of MRP2 was increased in cisplatin-resistant pancreatic cancer cells (Noma et al., 2008). Cui et al. evaluated that some specific genetic profiles of pancreatic cancer cells correlated with *in vitro* chemosensitivity. They found that inactivation of DPC4/SMAD4 (high-frequency pancreatic cancer driver genes) sensitized pancreatic cancer cells to cisplatin (Cui et al., 2012). Indeed, SUIT-2 cells do not contain mutation in the SMAD4 gene and thus are insensitive to cisplatin (Moore et al., 2001). Muralidharan-Chari et al. showed that exposure to chemotherapeutic drugs triggers pancreatic cancer cells to release micro-vesicles, which enable the expulsion of therapeutic drugs from human pancreatic cancer cells and from their microenvironment, contributing to their drug resistance (Muralidharan-Chari et al., 2016). Resistance to chemotherapy is a major problem in cancer treatment, therefore understanding the mechanism of drug resistance may lead to development of novel and effective therapeutics with the potential to sensitize pancreatic cancer cells to chemotherapy and improve anticancer efficacy of current treatments.

Nephrotoxicity is the major dose limiting side effect of cisplatin that causes acute (early) and chronic (advanced) kidney injury depending on the dosage, dose frequencies, and cumulative dose of cisplatin. A study of rats treated twice a week for 10 weeks with repeated lower doses of cisplatin 1 mg/kg (cumulative dose 20 mg/kg) with or without 100 mg/kg procainamide hydrochloride (cumulative dose 2 g/kg) showed protective activity against acute cisplatin-induced nephrotoxicity and hepatotoxicity (Fenoglio et al., 2005). Specifically, short‐duration and low‐volume hydration regimens with magnesium supplementation and mannitol are used in preventing cisplatin‐induced nephrotoxicity (Crona et al., 2017). Therefore, in our study mice were treated daily for 21 days with lower doses (1 mg/kg) of cisplatin supplemented with mannitol to prevent cisplatin-induced nephrotoxicity.

Recent studies have highlighted the role of cytokines in cancer drug resistance mechanisms and in cancer cell progression (Ho and Piquette-Miller, 2006; Jones et al., 2016). Observing changes in cytokine levels during chemotherapy may allow early diagnosis of cancer drug resistance. Increased cytokine levels in pancreatic cancer cells are most likely due to activation of the NF-κB pathway (Prabhu et al., 2014). Therefore, in our study the pharmacological inhibition of cisplatin-induced NF-κB activation by NDAT and XT199 may decrease the expression of pro-inflammatory cytokines IL-1β, TNF-α, IL-6 and increase anti-inflammatory cytokine IL-10. High levels of cytokines IL-1β, IL-6, IL-8, IL-10, TGF-β, and TNF-α are observed in patients with pancreatic cancer compared to healthy patients. Therefore, these cytokines are identified as novel candidate markers in the development and progression of pancreatic cancer (Dima et al., 2012; Blogowski et al., 2014; Yu and Kim, 2014). In addition, nerve injury increases the expression of inflammatory cytokines including IL-1β, IL-6, IL-17, and TNF-α and decreases the levels of anti-inflammatory/neuroprotective cytokines (IL-10, IL-4) that are involved in the pathogenesis of neuropathic pain (Fregnan et al., 2012; Lees et al., 2013; Janes et al., 2015).

Toll-like receptor (especially TLR4)/MyD88 dependent pathway leads to the activation of NF kB and subsequently the induction of pro-inflammatory cytokines (such as TNFα, IL-1β, IL-6) and reduction of anti-inflammatory cytokines (IL-10). This TLR4 signaling regulates chemotherapy-induced peripheral neuropathy in mice (Li et al., 2014; Park et al., 2014), and in the current work, cisplatin related chemotherapy-induced loss of motor dysfunction is ameliorated using αvβ3 integrin receptor antagonist to inhibit NF-kB activation and inflammation. Loss of motor function was not seen in untreated animals (control) implanted with pancreatic cancer cells (*i.e.*, loss of motor function is not due to pancreatic tumor).

Cytokines have a short half-life (generally <60 minutes) and are usually present at low concentrations in circulation (Sachdeva and Asthana, 2007). They act at hormonal concentrations with high receptor binding affinity of between 10^−12^ and 10^−10^ M. In some cases, only a few dozen receptors need to be activated per cell to elicit an effect. In vivo concentrations are in the range of ng/ml. Due to this local action at low concentrations, cytokine serum/plasma levels may not reliably reflect local activation (Sommer and Kress, 2004).

Initial sensory symptoms of cisplatin-induced peripheral neuropathy are numbness and paresthesias of the hind-limbs and then to the front-limbs in a “stocking-glove” distribution (Argyriou et al., 2014). Sensory nerve dysfunction is more common than motor involvement. However, motor neuropathy symptoms such as mild distal muscle weakness and atrophy due to denervation may also develop (Park et al., 2013). We observed no loss of motor function in animals receiving NDAT, XT199, or NDAT + cisplatin and XT199 + cisplatin. In contrast, cisplatin induced hind-limb spasticity after administration for 2 weeks, which is an indication of stocking-glove distribution caused by cisplatin-induced peripheral neuropathy. This provides evidence that NDAT and XT199 may preserve functional motor integrity by preserving nerve structure, which might be due to the modulation of cisplatin-induced upregulation of cytokines and chemokines by the αvβ3 antagonists NDAT and XT199.

## Materials and Methods

### Cell Culture and Preparation of Stock Solutions

Human pancreatic cancer SUIT2-luc cells (provided by Dr. Arumugam from MD Anderson Cancer Center, Houston, TX, USA) were grown in DMEM supplemented with 10% FBS and 1% penicillin/streptomycin. Cells were cultured at 37°C in a 5% CO_2_ humidified incubator to sub-confluence and treated with 0.25% (w/v) trypsin/EDTA to induce cell release from culture flasks. Cells were washed with culture medium, suspended in DMEM that was free of phenol red and FBS, and counted. Stock concentrations of test compounds were prepared in PBS (NDAT) and in DMSO (XT199) as 1 mM stocks for use in cellular assays. One mM stock solution of cisplatin was freshly dissolved in 0.9% sodium chloride and 10% D-mannitol added sterile water.

### Animals

Animal studies were conducted at the animal facility of the Veterans Affairs Medical Center, Albany, NY, USA and approved by the IACUC committee of the Veterans Affairs Medical Center. Immune-deficient female NCr-Foxn1 nude homozygous mice aged 5–6 weeks and weighing 18–20 g were purchased from Taconic Biosciences Inc. (Hudson, NY, USA). Mice were maintained under specific pathogen-free conditions and housed under controlled conditions of temperature (20–24°C), humidity (60–70%), and 12 h light/dark cycle with ad libitum access to water and food. Mice were allowed to acclimatize for 5 days before the study.

### Pancreatic Cancer Xenografts

For the development of orthotopic pancreatic tumor mouse models, SUIT2-luc cancer cells were harvested and injected (3 × 10^5^ cells per mouse, suspended in 30 μl of DMEM) into the pancreas of isoflurane anesthetized mice. Two days after tumor cell implantation, and immediately before the initiation of treatment, animals were randomized into 6 groups (5 animals/group) by tumor mass detected with an *in vivo* imaging system (IVIS^®^, Perkin Elmer, Boston, MA, USA) where average signal intensity was 0.25 E+07. Three animals were excluded from the randomization where tumor signal intensity was not detectable, *i.e.* < 0.05E+07. Numbers of animals in each treatment group are listed in **Table 1**. Treatments were administered subcutaneously (s.c.) or intraperitoneal (i.p.) daily for 21 days. Mice were observed daily during treatment for their hind-limb posture as an indication of acute pain response.

Orthotopic tumor growth was determined twice a week using IVIS^®^. Mice bearing SUIT2-luc tumors were anaesthetized using isoflurane and injected s.c. with 200 μl D-luciferin (30 mg/ml), then were imaged in the IVIS^®^. Photographic and luminescence images were taken at constant exposure time. Xenogen IVIS^®^ Living Image software version 4.5 was used to quantify non-saturated bioluminescence in regions of interest (ROI). Light emission between 5.5 × 10^6^–7.0 × 10^10^ photons was assumed to be indicative of viable luciferase-labeled tumor cells, while emissions below this range were considered as background. Bioluminescence was quantified as photons/second for each ROI. In vivo tumor kinetic growth and metastasis were monitored by signal intensity. After the termination of the study (day 21), animals were anaesthetized using isoflurane, sacrificed, and tumors were harvested for weighing and IVIS imaging.

### Tumor Histopathology

All tumors were analyzed with routine histopathology analysis. Tumors were fixed in 10% buffered formalin and tissues processed in a tissue processor (Tissue-Tek VIP, Miles Scientific, Newark, DE, USA) and then tissues were transferred into embedding chambers to hold them in position until the paraffin became solid to prevent further rotation. Once embedded, tissues were cut at 5 μm thickness on a microtome (Biocut 2030 Microtome, Leica, Buffalo Grove, IL, USA) onto charged glass slides. The sections were then deparaffinized and stained with hematoxylin and eosin (H&E staining protocol from imaging core facility at Oklahoma Medical Research Foundation). Sections were evaluated for various pathologic parameters using a light microscope (Leica EC3, Mag. 400X), and histopathological measurements of tumor area were performed in a double-blinded manner.

### Cytokine Assay

At animal sacrifice, blood was collected from hearts for analysis with a Bio-Plex^®^ 200 system assay. Plasma samples of 4 animals/group were used to analyze cytokine levels (IL-1β, IL-6, IL-10, TNF-α). The cytokine assay was performed strictly according to the manufacturer's protocol for plasma samples, utilizing recommended sample dilutions and standard curve concentrations, with all samples and standards assayed in duplicate.

### Statistical Analysis

Statistical analysis was performed using Student t-test as a parametric test and Kruskal-Wallis test as a non-parametric test and comparing the mean ± standard error of the mean (SEM) from each experimental group with its respective control group. Statistics were also evaluated using IBM SPSS Statistics 23.0. According to the Tukey HSD test, p values < 0.05 were considered significant.

## Conclusions

This study confirms that NDAT and XT199 exert anti-tumor and anti-inflammatory properties that reduce cisplatin resistance and alleviate cisplatin-mediated loss of motor function. Therefore, targeting several pathways by the αvβ3 antagonists NDAT and XT199 might overcome drug resistance and enhance the efficacy of cisplatin therapy in pancreatic cancer.

This study also provides an understanding of the role of NF-κB in pancreatic cancer, indicating that NF-κB may be an important therapeutic target for peripheral neuropathic complications. Further investigations are warranted to determine their efficacy in various tumor types with and without chemotherapy, which may have implications for improving the efficacy of systemic chemotherapy for patients with pancreatic cancer. To confirm our observational changes in motor function with NDAT and XT199 combination treatment with cisplatin in mice, future experiments should include quantitative sensory testing and histopathologic evaluation for evaluation of two important manifestations: axonal degeneration and demyelination after sciatic nerve injury in mice due to cisplatin treatment.

## Data Availability Statement

The datasets generated for this study are available on request to the corresponding author.

## Ethics Statement

Animal studies were conducted at the animal facility of the Veterans Affairs Medical Center, Albany, NY, USA and approved by the IACUC committee of the Veterans Affairs Medical Center.

## Author Contributions

Conceptualization: MD and SM. Methodology: MD, TS, DB, and SM. Validation: MD, TS, and SM. Formal analysis: MD, TS, and SM. Investigation: MD, TS, and SM. Resources: PD and SM. Data curation: MD, TS, and SM. Writing—original draft preparation: MD. Writing—review and editing: MD and SM. Visualization: MD and TS. Supervision: SC, PD, and SM. Project administration: MD and SM. Funding acquisition: SM.

## Funding

This work was supported by 2214-A International Doctoral Research Fellowship Programme of Scientific and Technological Research Council of Turkey (TÜBİTAK), Grant No: 1059B141401097 to MD and The Pharmaceutical Research Institute at Albany College of Pharmacy Health and Sciences, Rensselaer, NY, USA.

## Conflict of Interest

SM is an inventor in all patents and holds stock in a small pharmaceutical company, Nanopharmaceutical LLC, which is developing anticancer drugs, and PD is stock holder and Chief Scientific Officer at the company.

The remaining authors declare that the research was conducted in the absence of any commercial or financial relationships that could be construed as a potential conflict of interest.
